# Fibroblast Growth Factor 21 Promotes C2C12 Cells Myogenic Differentiation by Enhancing Cell Cycle Exit

**DOI:** 10.1155/2017/1648715

**Published:** 2017-10-04

**Authors:** Xinyi Liu, Yongliang Wang, Shuhong Zhao, Xinyun Li

**Affiliations:** ^1^Key Laboratory of Agricultural Animal Genetics, Breeding, and Reproduction of the Ministry of Education and Key Laboratory of Swine Genetics and Breeding of the Ministry of Agriculture, Huazhong Agricultural University, Wuhan 430070, China; ^2^The Cooperative Innovation Center for Sustainable Pig Production, Wuhan 430070, China

## Abstract

Fibroblast growth factor 21 (FGF21), a secretion protein, functions as a pivotal regulator of energy metabolism and is being considered as a therapeutic candidate in metabolic syndromes. However, the roles of FGF21 in myogenic differentiation and cell cycle remain obscure. In this study, we investigated the function of FGF21 in myogenesis and cell cycle exit using C2C12 cell line. Our data showed that the expression of myogenic genes as well as cell cycle exit genes was increased after* FGF21* overexpression, and* FGF21 *overexpression induces cell cycle arrest. Moreover, cell cycle genes were decreased in* FGF21 *overexpression cells while they were increased in* FGF21* knockdown cells. Further, FGF21/P53/p21/Cyclin-CDK has been suggested as the key pathway for cell cycle exit mediated by FGF21 in C2C12 cells. Also, we deduce that FGF21 promotes the initiation of myogenic differentiation mainly through enhancing cell cycle exit of C2C12 cells. Taken together, our results demonstrated that FGF21 promotes cell cycle exit and enhances myogenic differentiation of C2C12 cells. This study provided new evidence that FGF21 promotes myogenic differentiation, which could be useful for better understanding the roles of FGF21 in myogenesis.

## 1. Introduction

Fibroblast growth factor 21 (FGF21), a member of the FGF family, was initially identified by Nobuyuki Itoh's group [1], and its bioactivity was first discovered as a potential regulator of glucose uptake in mouse and human adipocytes [2]. Signaling pathway studies indicated that FGF21 could bind fibroblast growth factor receptor (FGFR) 1/2 and coreceptor *β*-Klotho after being secreted from tissues [3, 4]. FGFR1 or *β*-Klotho knockout mice are refractory to the effects mediated by FGF21 [5–7]. FGF21 can be induced in multiple tissues including liver, pancreas, adipose tissue, and skeletal muscle [8–12]. However, the major organ of FGF21 releasing varies in response to different stimulation. In rodents, the liver tissue is the major source of FGF21 in the conditions of fasting and ketogenesis [13, 14], while under cold exposure, brown adipose tissue becomes an important organ of FGF21 generation [15]. In addition, circulating FGF21 was also increased in several pathological statuses including obesity, diabetes, and other metabolic syndromes in rodents and humans [16–18]. As reported, in obesity and diabetes, markedly elevated circulating FGF21 level has led to an FGF21-resistant state. Moreover, the response to exogenous FGF21 is impaired and the expression of FGF21 receptors is reduced [19–21].

It has been demonstrated that FGF21 functions as a regulator of glucose and lipid metabolism as well as energy homeostasis. Some studies reported that increasing of FGF21 could ameliorate obesity and diabetes through enhancing glucose uptake, suppressing adipogenesis, or increasing lipolysis both in vitro and in vivo [22–24]. Currently, FGF21 has been treated as a therapeutic target of metabolism disorders [25]. Furthermore, FGF21 can prevent isoproterenol-induced cardiac hypertrophy and diabetes-induced cardiac apoptosis through MAPK pathway in mice [26, 27]. FGF21 also plays important roles in myogenesis. In the first few years, skeletal muscle was not considered as a source of FGF21. Later, one study carried out on mice demonstrated that skeletal muscle can also act as a source of FGF21 and it can be regulated via PI3K/Akt1 pathway [12]. Moreover, some studies reported that FGF21 could be induced in skeletal muscle by hyperinsulinemia and exercise in human beings [28–31]. FGF21 is associated with myogenic differentiation and regulated by myogenic factor MyoD; also, FGF21 is involved in myofiber type transformation [32, 33].

FGF21 is currently considered as a myokine and adipokine, which mediates the cross talk between skeletal muscle and adipose tissue in energy metabolism [34, 35]. In human skeletal muscle, FGF21 can directly increase glucose uptake [17]. It has been reported that the improvement of glucose clearance by FGF21 treatment was lost in the absence of UCP1 [36]. Furthermore, a study revealed that the ectopic expression of UCP1 in skeletal muscle upregulates FGF21 level in both skeletal muscle and circulation system, and increased FGF21 promotes browning of white adipose tissue [35]. In addition, FGF21 enhances mitochondrial function via PGC1*α* pathway in response to skeletal muscle mitochondrial respiratory chain deficiency in humans. Further, lack of FGF21 impairs the upregulation of PGC1*α* induced by fasting in mice [37–39].

Previous studies confirmed that FGF21 plays important roles in myogenesis. In order to further investigate the mechanisms of FGF21 in myogenesis, we first detected the expression of FGF21, *β*-Klotho, MyoD, Myogenin, Myf5, and cell cycle related genes during myogenic differentiation of C2C12 cells. Then, the function and mechanisms of FGF21 in myogenesis and cell cycle progression were studied in vitro. Our study offered useful information for better understanding the roles of FGF21 in myogenesis.

## 2. Materials and Methods 

### 2.1. Cell Culture and Differentiation

C2C12 mouse myoblasts were cultured in DMEM containing 10% FBS and maintained in a humidified incubator with 5% CO_2_ at 37°C. For myogenic differentiation, when confluence was reached, C2C12 cells were shifted into a differentiation medium supplemented DMEM with 2% FBS.

### 2.2. FGF21 Overexpression and Stable Overexpression Cells Selection

The full length of mouse FGF21 CDS sequence was amplified using gene-specific primers and cloned into pCDNA3.1 expression vector. After being verified by sequencing, the expression vector containing full length of mouse FGF21 CDS was named pCDNA3.1-FGF21. For FGF21 overexpression, C2C12 cells were seeded on a 6-well plate the day before; when cells reached 60–70% confluence, transfection of FGF21 overexpression vector (pCDNA3.1-FGF21) and control vector (pCDNA3.1) was carried out using Lipofectamine 2000 following the manufacturer's instructions. In terms of establishment of FGF21 stably expressed cells, 48 hours after transfection, cells were selected by adding G418 to the culture medium. After at least 2 weeks of selection, FGF21 stably expressed cells were obtained.

### 2.3. RNA Interference

In order to knock down FGF21 gene, double-stranded small interference RNAs targeting FGF21 and scrambled sequence as control were obtained from GenePharma (Shanghai, China). C2C12 cells were transfected with siRNAs, and after 48 hours, cells were harvested for analysis.

### 2.4. Enzyme-Linked Immunosorbent Assay (ELISA)

Cell medium was collected at 0 and 2 days of myogenic differentiation individually and centrifuged at 3000 rpm for 5 minutes to harvest the cell supernatant. The level of FGF21 protein in the cell supernatant was detected using the mouse FGF21 ELISA kit (Hangzhou, China) according to the manufacturer's instructions.

### 2.5. RNA Extraction, Reverse Transcription, and Quantitative PCR

Total RNA was isolated with TRIzol reagent (Invitrogen, USA) following the manufacturer's instructions. The purity and concentration of the RNA samples were measured using NanoDrop 2000. After that, 1 microgram of each RNA sample was used to synthesize cDNA using PrimeScript 1st strand cDNA synthesis kit (Takara, Japan) following the manufacturer's instructions. Real-time quantitative PCR was performed using QuantiFast SYBR Green PCR kit (Qiagen, USA) on a CFX96 qPCR System (Bio-Rad, USA). And mRNA levels were quantified relatively to the expression of GAPDH by employing 2^−ΔΔct^ value, and Student's *t*-test was used for statistical comparison. Primers for quantitative PCR are listed in the Supplement Table (in the Supplementary Material available online at https://doi.org/10.1155/2017/1648715).

### 2.6. Western Blot

Total protein was extracted using RIPA buffer and the concentration was determined by Pierce BCA protein assay kit (Thermo, USA). Equal amounts of proteins were loaded and separated by SDS-PAGE gel before being blotted onto PVDF membranes (Millipore, USA). After the transfer, the membranes were blocked by 5% BSA for 1 hour at room temperature and then incubated with a primary antibody at 4°C overnight. The antibodies used in this study include FGF21 (1 : 1000, Abcam, USA), MyoD (1 : 500, Santa Cruz, USA), MyoG (1 : 500, Santa Cruz, USA), MEF2c (1 : 500, Santa Cruz, USA), MHC (1 : 500, Santa Cruz, USA), CDK4 (1 : 1000, CST, USA), P21 (1 : 1000, R&D, USA), P53 (CST) Cyclin D1 (1 : 1000, R&D, USA), Cyclin D3 (1 : 1000, R&D, USA), and tubulin (1 : 1000, Abcam, USA), which were diluted with 5% BSA.

### 2.7. Immunofluorescence

For immunofluorescence analysis, after differentiation, cells were washed with cold PBS and then fixed in 4% paraformaldehyde for 40 minutes and permeabilized in 0.2% Triton X-100-PBS for 10 minutes. After washing three times (10 minutes each) in PBS, cells were blocked with 5% BSA for 2 hours at room temperature and then incubated with anti-MHC primary antibody (Santa Cruz) at 4°C overnight. Alexa Fluor 488-labeled goat anti-mice IgG (Beyotime, China) was used as secondary antibody, and nuclei were stained with DAPI. The fluorescence images were gathered using Axio Observer microscope (Zeiss, USA) and Image Pro Plus.

### 2.8. Cell Cycle Analysis

The FGF21 gene stable overexpression cells were seeded on a 6-well plate and cultured for 24 hours; cells were then washed with PBS and fixed in 70% ethanol at 4°C overnight. Subsequently, cells were resuspended with PBS and incubated with propidium iodide (PI) in the presence of RNase A at 37°C for 30 minutes, and then DNA content was analyzed using a flow cytometer (Beckman, USA).

## 3. Results

### 3.1. FGF21 Showed Similar Expression Pattern to Myogenic Marker Genes and Cell Cycle Exit Genes during Myogenic Differentiation

**Figure 1:**
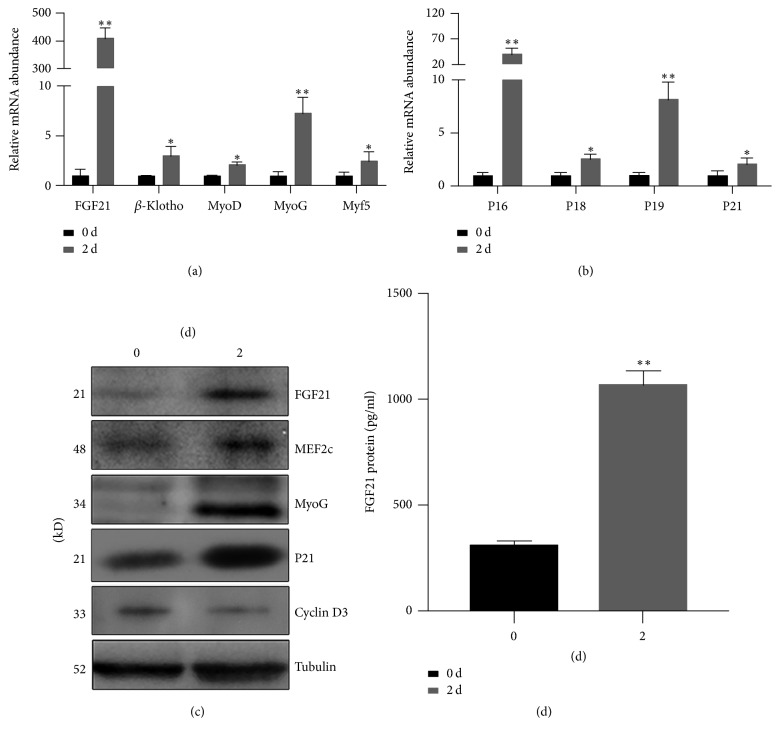
The expression of FGF21 and cell cycle exit genes was increased in the early stage of myogenic differentiation. (a) C2C12 myoblast cells were differentiated for 0 and 2 days, and mRNA levels of FGF21 and myogenic differentiation key genes, MyoG, MyoD, and Myf5, were measured using Q-PCR. (b) The mRNA expression levels of cell cycle inhibitor genes were determined in C2C12 cells after being differentiated for 0 and 2 days by Q-PCR. (c) Western blot was applied to detect the changes in protein level of FGF21, myogenesis genes, and cell cycle related genes. (d) FGF21 protein level in cell medium at 0- and 2-day differentiation was measured by ELISA. GAPDH and tubulin were used as internal control for Q-PCR and western blot, respectively. The expression level of genes at day 0 was set as 1. All the data are presented as the mean ± SD (*n* ≥ 3). ^*∗*^*P* < 0.05, ^*∗∗*^*P* < 0.01.

First, we assessed the gene expression patterns at the early stage of myogenic differentiation of C2C12 cells. As is shown, the expression of FGF21 and its coreceptor *β*-Klotho as well as myogenic factors MyoD, MyoG, and Myf5 was increased at the transcriptional level in response to early myogenic differentiation (Figure 1(a)). Further, the mRNA levels of cell cycle exit genes P16, P18, P19, and P21 were upregulated during early myogenic differentiation (Figure 1(b)). Moreover, western blot results confirmed that FGF21, MEF2c, MyoG, and P21 were upregulated during the early stage of myogenic differentiation, while Cyclin D3 was downregulated (Figure 1(c)). In addition, the ELISA results demonstrated that the FGF21 protein level in the cell medium is also increased in response to myogenic differentiation (Figure 1(d)).

### 3.2. Overexpression of FGF21 Promotes Myogenic Differentiation and Inhibits Proliferation of C2C12 Cells

**Figure 2:**
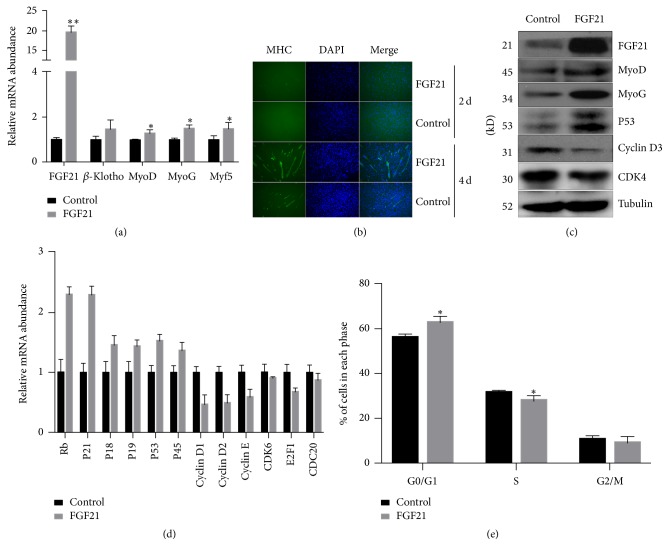
Myogenesis was enhanced while cell cycle was suppressed in FGF21 stable expression myoblasts. (a) The mRNA expression levels of myogenic differentiation determination genes were detected in FGF21 stably transfected C2C12 cells. (b) Immunofluorescence of MHC in FGF21 overexpression or control C2C12 cells after 2 and 4 days of differentiation. (c) The protein levels of genes involved in myogenic differentiation as well as cell cycle were determined by western blot in FGF21 overexpression cells. (d) The mRNA levels of cell cycle related genes were determined by Q-PCR in FGF21 overexpression or control C2C12 cells. (e) Quantification of cell cycle distribution of FGF21 stably transfected cells analyzed by flow cytometry. GAPDH and tubulin were used as internal control for Q-PCR and western blot, respectively. For Q-PCR, the expression level of genes in the control group was set as 1. All the data are presented as the mean ± SD (*n* ≥ 3). ^*∗*^*P* < 0.05, ^*∗∗*^*P* < 0.01.

In order to elucidate the functions of FGF21 in myogenic differentiation, FGF21 stable overexpression C2C12 cells were established. Q-PCR results showed that the mRNA levels of MyoG, MyoD, and Myf5 were significantly upregulated in FGF21 overexpression cells (*P* < 0.05). The expression of *β*-Klotho has a tendency of upregulation in FGF21 overexpression cells but did not reach a statistically significant level (Figure 2(a)). Also, immunofluorescence results showed that MHC signal at 4 days of differentiation was obviously stronger in FGF21 overexpression C2C12 cells (Figure 2(b)). In addition, western blot results also showed that MyoD and MyoG were elevated at protein level in FGF21 overexpression cells (Figure 2(c)). Further, P53, a cell cycle inhibiting gene, was increased, while cell cycle genes Cyclin D3 and CDK4 were decreased in response to elevated FGF21 (Figure 2(c)). In addition, mRNA levels of cell cycle exit related genes, Rb, P21, P18, P19, P53, and P45, exhibited a significant increase; in contrast, the cell cycle genes, Cyclin D1, Cyclin D2, Cyclin E, CDK6, E2F1, and CDC20, were significantly decreased in FGF21 overexpression group (Figure 2(d)). Moreover, flow cytometry results showed that FGF21 overexpression significantly increased the percentage of cells in G1 phase whereas the percentage of cells in S phase was significantly decreased when compared with control (Figure 2(e)).

### 3.3. FGF21 Knockdown Attenuated Cell Cycle Exit Process

**Figure 3:**
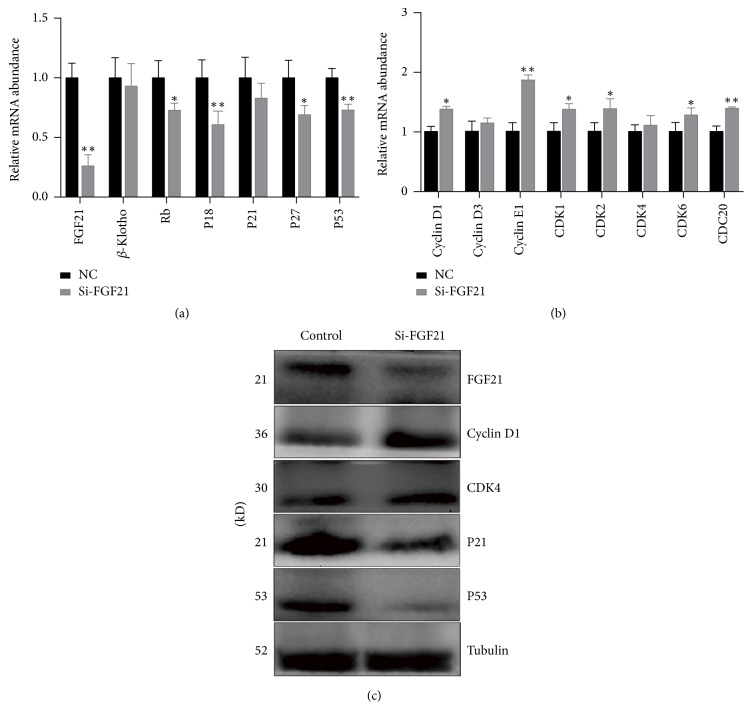
FGF21 knockdown attenuated the suppression of cell cycle genes mediated by FGF21. (a, b) C2C12 cells were transfected with FGF21 siRNA or scramble control, and then the mRNA levels of cell cycle exit genes (a) and cell cycle genes (b) were detected by Q-PCR. (c) Proteins involved in cell cycle were analyzed by western blot. ^*∗*^*P* < 0.05; ^*∗∗*^*P* < 0.01.

To further investigate the role of FGF21 in cell cycle exit, we performed FGF21 knockdown in C2C12 cells using siRNA. The mRNA expression levels of cell cycle exit genes Rb, P18, P21, P27, and P53 were all significantly decreased in FGF21 knockdown (Si-FGF21) group. The mRNA level of *β*-Klotho showed no significant change after FGF21 knockdown (Figure 3(a)). In contrast to cell cycle exit genes, the mRNA levels of cell cycle genes were increased in response to FGF21 gene suppression (Figure 3(b)). Western blot results showed that the cell cycle genes Cyclin D1 and CDK4 were elevated, whereas the protein levels of cell cycle exit genes P21 and P53 were repressed in FGF21 knockdown group (Figure 3(c)).

## 4. Discussion

**Figure 4:**
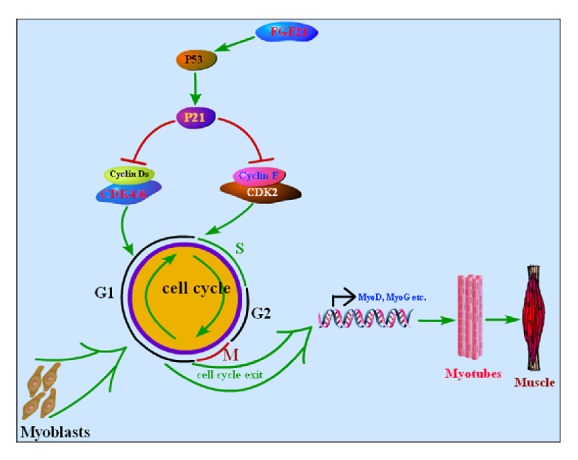
Possible mechanism of FGF21 promoting cell cycle exit and enhancing myogenesis. FGF21 upregulates the expression of P53, P53 increases P21, and then the increased P21 inhibits Cyclin/CDK complexes and arrests cells at G1 phase of cell cycle, and thus myogenic differentiation was enhanced due to cell cycle exit of myoblasts.

FGF21 is a pleiotropic secretion protein, which possesses multiple metabolic benefits including ameliorating insulin resistance, improving hepatic lipid accumulation, and extending lifespan [24, 40–42]. In the past decades, the bulk of scientific research focused on the mechanism of FGF21 regulating glucose and lipid metabolism in obesity and diabetes. Lots of breakthroughs were made and FGF21 was considered as a drug candidate for some metabolic syndromes. In this study, we analyzed the effect of FGF21 on cell cycle progression and myogenic differentiation. Our results demonstrated that FGF21 downregulates the expression of cell cycle genes while it upregulates the expression of cell cycle inhibiting genes and myogenic genes, which was consistent with the point that, in response to myogenic differentiation, cell cycle exit genes were induced and cells were promoted to withdraw from cell cycle irreversibly [43–45]. In addition, we found a probable pathway where FGF21 enhances myogenic differentiation through promoting cell cycle exit (Figure 4).

Myogenic differentiation is accompanied with increasing energy requirement and ATP level rise in the process of myoblasts differentiation into myotubes [46, 47]. In this study, the secretion of FGF21 is dramatically increased in response to myogenic differentiation. Also, FGF21 was reported to enhance glucose uptake in human skeletal muscle directly or via Glut1 and Glut4 [17, 48]. Further, FGF21 can promote mitochondrial function via upregulating the expression of PGC1*α* [34, 37]. Therefore, we deduce that elevated FGF21 plays crucial roles in energy generation to adjust increasing energy demand during myogenic differentiation.

It was suggested that FGF21 functions via combination to its receptor and *β*-Klotho complex [49, 50]. Skeletal muscle was not considered as an FGF21 signal target in the first few years due to the lack of *β*-Klotho expression [51]. However, more and more studies confirmed that FGF21 can be secreted from skeletal muscle and can target this tissue [31, 33, 52]. Moreover, it has been reported that *β*-Klotho can be induced in myotubes in humans [48, 53]. In addition, one study suggested that FGF21 signal transduction can be performed in a *β*-Klotho independent pathway [54]. In this study, the expression of *β*-Klotho was increased in response to myogenic differentiation but did not change significantly in FGF21 overexpression and knockdown cells. Thus, *β*-Klotho may not be regulated by FGF21 and FGF21 may function either in a *β*-Klotho dependent or a *β*-Klotho independent manner in skeletal muscle.

Our previous study indicated that FGF21 could induce myogenesis [33], while the molecular mechanisms remain obscure. It is well known that P21 is a main inhibitor of cell cycle progression, and P21 can be transcriptionally activated by P53 [55]. Also, Cyclin Ds/CDK4 and CDK6 and Cyclin E/CDK2 complexes function in G1 phase and G1/S transition [56, 57]. P21 not only inhibits Cyclin Ds/CDK4 and CDK6 complexes but also inhibits Cyclin Es/CDK2 complexes to arrest cell proliferation. Therefore, we proposed that FGF21 upregulates P53, elevated P53 transcriptionally activates P21, and P21 inhibits Cyclin/CDK complexes, which leads to G1 phase arrest and enhances myogenesis of myoblast cells.

In this study, we confirmed that, as expected, FGF21 is upregulated during myogenic differentiation of myoblasts. In addition, we showed that FGF21 could promote myogenic differentiation of myoblasts via enhancing cell cycle exit of myoblasts. Also, FGF21/P53/p21/Cyclin-CDK has been suggested as the key pathway for cell cycle exit mediated by FGF21 in C2C12. Taken together, our study provided new clue to better understand the roles of FGF21 in myogenesis.

## Supplementary Material

Supplement Table 1. Primers for quantitative PCR.

## Abbreviations

FGF21: Fibroblast growth factor 21
MyoG: Myogenin
CDKs: Cyclin-dependent kinases
MAPK: Mitogen-activated protein kinase
PI3K: Phosphoinositide 3-kinase
UCP1: Uncoupling protein 1
PGC1*α*: Peroxisome proliferator-activated receptor gamma coactivator 1-alpha.
