# Supplementary material for: Fibroblast Growth Factor 21 Promotes C2C12 Cells Myogenic Differentiation by Enhancing Cell Cycle Exit
Source: Biomed Res Int. 2017 Oct 4;2017:1648715. doi: 10.1155/2017/1648715 (PMC5646352; doi:10.1155/2017/1648715)
Supplement: Supplementary file 1 — Supplement Table 1. Primers for quantitative PCR. [file 1648715.f1.pdf]

Supplement Table. Quantitative PCR primer sequences

Gene	Forward	Reverse	Product size (bp)
FGF21	GTGTCAAAGCCTCTAGGTTTCTT	GGTACACATTGTAACCGTCCTC	123
b-Klotho	TCGGTACGTCTACTCACACCT	AGCCAACAAGTCTTTTTCCAGA	88
MyoD	ATGATGACCCGTGTTTCGACT	CACCGCAGTAGGGAAGTGT	119
MyoG	GCAGGCTCAAGAAAGTGAATGA	TAGGCGCTCAATGTACTGGAT	122
Myf5	GCCTTCGGAGCACACAAAG	TGACCTTCTTCAGGCGTCTAC	187
Rb	TTGGAGTCCGATTGTATTACCGT	AGCACAGGCCAGTAAAGACAT	128
P16	CGCAGGTTCTTGGTCACTGT	TGTTCACGAAAGCCAGAGCG	127
P18	GGGGACCTAGAGCAACTTACT	AAATTGGGATTAGCACCTCTGAG	149
P19	CTGGAAGAAGTCTGCGTCGG	GTCTTGCCAAAGCGGTTCAG	125
P21	CGAGAACGGTGGAACTTTGAC	CCAGGGCTCAGGTAGACCTT	107
P27	TCAAACGTGAGAGTGTCTAACG	CCGGGCCGAAGAGATTTCTG	103
P45	CCTCCAAGGAAACGAGTCAAG	CAGGAGACACCTGGAAAGTTC	98
P57	GCAGGACGAGAATCAAGAGCA	GCTTGGCGAAGAAGTCGTT	119
CyclinD1	TGACTGCCGAGAAGTTGTGC	CTCATCCGCCTCTGGCATT	164
CyclinD2	GAGTGGGAACTGGTAGTGTTG	CGCACAGAGCGATGAAGGT	154
CyclinD3	TGGATCGCTACCTGTCCTG	CCTGGTCCGTATAGATGCAAAG	138
cyclin E1	CCGTCTTGAATTGGGGCAATA	GAGCTTATAGACTTCGCACACC	167
CDK1	AAATCCTCCAGGGAATTGTGTTT	CAGCCAGTTTGATTGTTCCTTTG	105
CDK2	ATGGAGAACTTCCAAAAGGTGG	CAGTCTCAGTGTCGAGCCG	124
CDK4	AAGGTCACCCTAGTGTTTGAGC	CCGCTTAGAAACTGACGCATTAG	119
CDK6	TCTCACAGAGTAGTGCATCGT	CGAGGTAAGGGCCATCTGAAAA	123
E2F1	TGCAGAAACGGCGCATCTAT	CCGCTTACCAATCCCCACC	122
CDC20	TTCGTGTTCGAGAGCGATTTG	ACCTTGGAACTAGATTTGCCAG	191
GAPDH	AGGTCGGTGTGAACGGATTTG	TGTAGACCATGTAGTTGAGGTCA	123
